# Strain-specific pathogenicity and antibody-dependent enhancement of dengue virus infection in Ifnar1–Ifngr1 double-knockout mice

**DOI:** 10.1099/jgv.0.002267

**Published:** 2026-05-18

**Keywords:** antibody-dependent enhancement (ADE), dengue virus (DENV), *Ifnar1–Ifngr1 *double-knockout (DKO) mice, mouse model

## Abstract

This study investigated the pathogenicity of various dengue virus (DENV) strains using a newly established double-knockout (DKO) mouse model lacking both type I and type II IFN receptors (*Ifnar1–Ifngr1* DKO), rendering the mice highly susceptible to DENV infection. Survival, body weight changes and histopathological outcomes were assessed to characterize strain-specific pathogenicity and the potential for antibody-dependent enhancement (ADE). We observed significant strain-dependent variation in virulence. Notably, certain strains, such as DENV-1 02-20, exhibited pronounced ADE, evidenced by increased mortality and weight loss following pre-administration of the anti-DENV monoclonal antibody 4G2. These effects were more severe than those observed in single-knockout mice (*Ifnar1* KO or *Ifngr1* KO) and in control groups. Histopathological examination revealed necrotic lesions in the brain and spinal cord, particularly in the olfactory bulb and cerebral cortex, along with extensive apoptosis of splenic lymphocytes, indicating severe immune activation and tissue damage. Our findings demonstrate that this lethal dengue infection model using *Ifnar1–Ifngr1* DKO mice is an effective and valuable platform for studying the complex dynamics of DENV pathogenesis, immune responses and antibody interactions, as well as for evaluating vaccines and therapeutic strategies aimed at improving future prevention and treatment strategies for DENV infection.

**Impact Statement**

DENV is a significant global health concern, causing millions of infections each year, yet there are still limited options for treatment and prevention. This study presents a type I and type II IFN receptor double-knockout mouse model, developed on the commonly used C57BL/6J genetic background, providing a cost-effective and reliable system for studying the pathogenic mechanisms of DENV strains and their immune responses. It also allows the study of antibody-dependent enhancement, a phenomenon where pre-existing antibodies exacerbate viral infection, which is important for understanding vaccine safety. Rather than recapitulating the full clinical spectrum of human dengue infection, this model captures key virological and immunopathological features associated with severe disease – such as viraemia, immune system dysregulation and viral dissemination to the nervous system – offering a valuable tool for scientists developing vaccines, treatments and strategies to reduce the global burden of dengue.

## Data Summary

The authors confirm that all supporting data and protocols have been provided within the article.

## Introduction

Dengue virus (DENV) remains a major global health concern, predominantly affecting tropical and subtropical regions. Since early 2023, the virus has been spreading persistently, with unexpected surges in dengue fever cases leading to record-high numbers of infections and deaths reported in more than 80 countries across all 5 WHO regions: Africa, the Americas, Southeast Asia, the Western Pacific and the Eastern Mediterranean [1]. DENV causes an acute febrile illness known as dengue fever, which can progress to life-threatening complications such as dengue haemorrhagic fever and dengue shock syndrome [2]. Currently, no specific antiviral treatment for DENV exists, and vaccine development – the only preventive measure – still faces challenges [3,5]. Therefore, research aimed at elucidating the pathogenesis of DENV infection, and the host immune responses is essential for developing effective vaccines and treatments [6,7]. In particular, animal models that enable the investigation of severe and dysregulated DENV infection – which represents a minority but clinically critical outcome in humans – are crucial for evaluating viral pathogenicity and therapeutic efficacy [7].

As DENV is a mosquito-borne virus that primarily infects humans, limited animal models that can naturally replicate DENV infections are available. Traditionally, non-human primate and certain rodent models have been used in research, but they do not fully recapitulate the severe symptoms observed in humans [7,8]. This has prompted the development of animal models that more accurately replicate human disease pathology. Consequently, mouse models, particularly those with specific immune deficiencies, have gained attention and are increasingly utilized in DENV research [8,10].

IFN receptors play a crucial role in initiating early immune responses against viral infections. Specifically, type I IFN (IFN-α/β) signalling suppresses viral replication. In the absence of IFN signalling, viruses can replicate more easily, leading to widespread infection [11,12]. Leveraging this mechanism, *Ifnar1*-knockout (KO) mice (*Ifnar1* KO), which lack type I IFN signalling, have been used as DENV infection models. These mice are highly susceptible to DENV and allow effective evaluation of disease progression and viral pathogenicity, capturing several features relevant to human infection [10,13, 14].

*Ifnar1* and *Ifngr1* double-knockout (DKO) mice (*Ifnar1–Ifngr1* DKO mice), such as the commonly used AG129 mice, lack both type I and type II [IFN-gamma (IFN-γ)] IFN signalling and have been established as highly susceptible hosts for DENV infection [15]. Compared to *Ifnar1*-KO mice, DKO mice exhibit more severe disease manifestations, providing a valuable platform for investigating viral pathogenesis and host immune responses. Moreover, this model has been widely used to study the virulence of different DENV strains, evaluate vaccine efficacy and examine antibody-dependent enhancement (ADE) – a phenomenon in which antibodies enhance rather than neutralize the infection, thereby contributing to severe disease and complicating vaccine development [16,19]. Studies using both *Ifnar1*-KO and *Ifnar1–Ifngr1* DKO mice have confirmed that ADE occurs with specific DENV strains, underscoring the complex role of antibodies in DENV infection [19,21]. Therefore, *Ifnar1–Ifngr1* DKO mice serve as a robust platform for comparing pathogenicity across DENV strains and play an indispensable role in the preclinical evaluation of vaccines and therapeutic agents [22].

Animal models are critical for elucidating the pathogenesis and immune mechanisms underlying DENV infections. Although the AG129 mouse model has been widely used owing to its high susceptibility, it has notable limitations, including reliance on mouse-adapted DENV strains, limited representation of human clinical disease, limited commercial availability and high licensing costs [23,24]. Furthermore, since the introduction of the AG129 mouse model in 1995, few cost-effective and highly susceptible alternatives on the commonly used C57BL/6 mouse strain background have been developed [23]. Compared with the AG129 model, which is maintained on a 129/Sv background, the *Ifnar1–Ifngr1* DKO mice used here are on a C57BL/6J background, allowing direct integration with a wide range of immunological and genetic tools. Therefore, in this study, we aimed to establish a highly susceptible C57BL/6J *Ifnar1–Ifngr1* DKO mouse model that enables robust analysis of DENV ADE, pathogenesis and immune responses, providing a valuable platform for vaccine and therapeutic development.

## Methods

### Animals

**Fig. 1.**
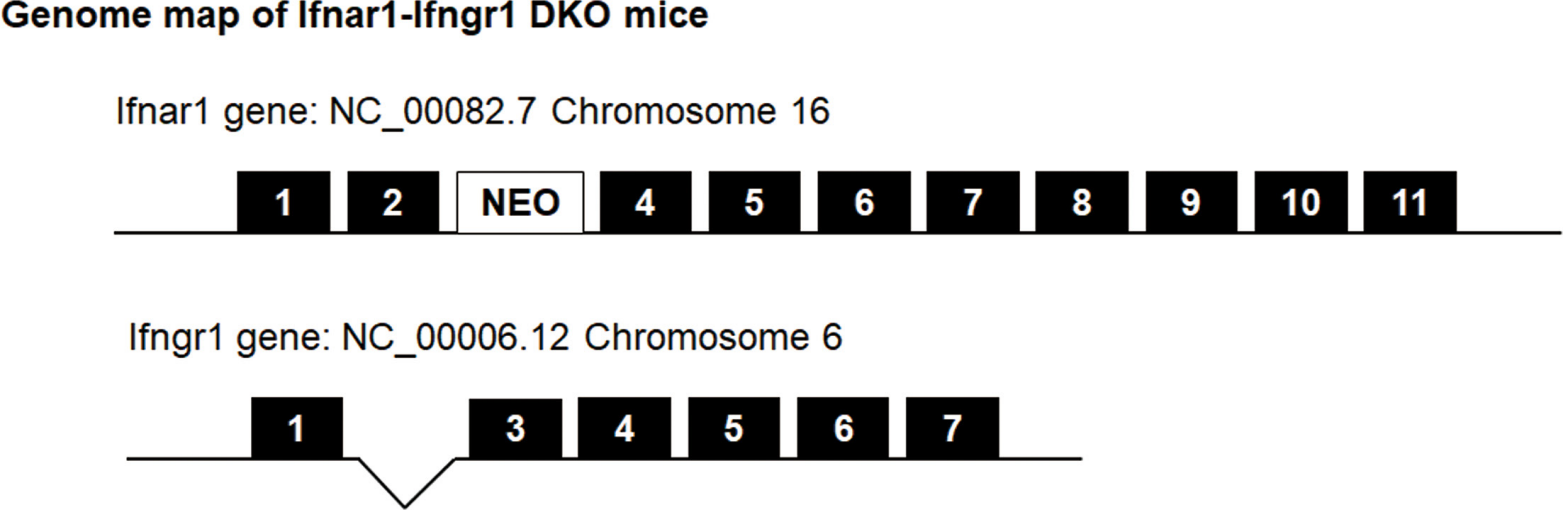
Genetic schematic of IFN receptor DKO (*Ifnar1–Ifngr1* DKO) mice. Diagram illustrating the disrupted alleles for the *Ifnar1* and *Ifngr1* genes in the DKO mouse line used in this study.

Given the critical role of *Ifnar1–Ifngr1* DKO mice in DENV research, we established these mice to facilitate our study. The *Ifngr1*-KO mice were generated by deleting exon 2 (115 bp) of the *Ifngr1* gene using the CRISPR/Cas9 gene editing system. This modification was performed by Unitech Co. Ltd. (Chiba, Japan). The *Ifnar1*-KO mice were produced by interbreeding heterozygous mice obtained from crossing B6.129-*Dnase2a*<tm1Osa> *Ifnar1*<tm1Agt> mice [25] (Riken BioResource Center, Ibaraki, Japan) with C57BL/6J mice (Japan SLC, Inc., Shizuoka, Japan). The heterozygous offspring were then intercrossed to obtain homozygous *Ifnar1*-KO mice. To establish the *Ifnar1–Ifngr1* DKO mice, we crossed the *Ifnar1*-KO and *Ifngr1*-KO mice. The genetic map of the DKO mice is shown in Fig. 1. The DKO genotype was verified via PCR using tail samples collected at 3 weeks of age. The primer sequences and amplicon sizes for the *Ifnar1*-KO mice were obtained from the RIKEN Strain Data Sheet (RBRC04021) available from the RIKEN BRC web catalogue (https://mus.brc.riken.jp/en/search_for_mouse_strain). For the *Ifngr1* locus, PCR with primers AAAGAGAGCATAGCGGGAAATAC and CTTAGAAGAAAACGCAATGGACA produced amplicons of 1.1 kbp in *Ifngr1*-KO mice and 2.3 kbp in C57BL/6J mice.

### Viral strains and p.f.u. determination

The DENV strains used in this study were DENV-1 (02–20, GenBank accession no. AB178040), DENV-1 (D1/Hu/Saitama/NIID100/2014, GenBank accession no. LC011945), DENV-2 (S16803, GenBank accession no. GU289914), DENV-2 (09-74, GenBank accession no. LC367234), DENV-2 [New Guinea C (NGC); ATCC VR-1584], DENV-3 (CH53489, GenBank accession no. DQ863638), DENV-3 (00-40, GenBank accession no. AB111082), DENV-4 (TVP/360, GenBank accession no. KU513442), DENV-4 (09-48, GenBank accession no. LC069810) and DENV-4 (H241; ATCC VR-1490).

DENV was propagated in African green monkey kidney cells (Vero; JCRB9013). Virus titres were determined by plaque assay. Vero cells were seeded in 12-well plates at a density of 2×10^5^ cells/well. The following day, cells were infected with serial dilutions of virus suspension and incubated for 1 h at 37 °C. After infection, the inoculum was removed, and cells were overlaid with minimum essential medium containing 2% FBS and 1% methylcellulose. After 5 days of incubation, cells were fixed with formalin for 1 h at room temperature. Plaques were visualized by crystal violet staining, and virus titres were calculated as p.f.u. per millilitre.

### Animal experiments and viral quantification

*Ifnar1–Ifngr1* DKO mice aged 7–16 weeks were intraperitoneally inoculated with 250 µl of DENV at doses ranging from 1×10^1^ to 1×10^6^ p.f.u. for LD₅₀ determination, depending on the viral strain. For ADE evaluation and comparative pathogenicity analyses, higher viral doses (1×10⁷ p.f.u.) were used and were not included in LD₅₀ calculations. To evaluate the influence of antibodies on disease progression, a subset of mice was administered the anti-DENV monoclonal antibody 4G2 (clone 4G2; mouse IgG2a), originally generated in mice and obtained from ATCC (HB-112), at a dose of 50 µg per mouse in 100 µl PBS 1 day prior to viral inoculation. The dose was selected based on previous ADE studies demonstrating robust enhancement at comparable antibody concentrations [26,27]. Control mice received PBS alone. Mice were euthanized when body weight loss exceeded 25% of the initial body weight, in accordance with the approved animal ethics protocol.

Mice were euthanized under isoflurane anaesthesia at 21 days post-infection (dpi) or at specific time points (1, 3, 5, 7, 10 and 14 dpi). Blood samples were collected from infected mice, and total RNA was extracted from serum using the MagMAX Pathogen RNA/DNA Kit (Thermo Fisher Scientific, Waltham, MA, USA), following the manufacturers’ instructions.

Viral genome quantification was performed using quantitative reverse transcription PCR (qRT-PCR) with the THUNDERBIRD Probe One-Step qRT-PCR Kit (Toyobo, Osaka, Japan) and TaqMan probes, as previously described [28]. Briefly, reverse transcription was performed at 50 °C for 10 min, followed by initial denaturation at 95 °C for 1 min. PCR amplification consisted of 40 cycles at 95 °C for 10 s and 60 °C for 30 s. All data were acquired and analysed using the LightCycler 480 II system (Roche, Vienna, Austria).

### Histopathological analysis of infected mice

Tissue samples collected at 1, 3, 5, 7, 10 and 14 dpi were fixed in 10% phosphate-buffered formalin. These include the brain, heart, lung, pulmonary lymph nodes, trachea, liver and kidney, including adrenal gland, spleen, pancreas, stomach, small intestine, large intestine, mesenteric lymph nodes, bladder, spinal cord and femoral bone marrow. The fixed tissues were embedded in paraffin, sectioned and stained with haematoxylin and eosin (HE). Immunohistochemical staining was performed on the paraffin-embedded sections using a rabbit polyclonal anti-DENV NS3 protein antibody (1 : 500; GeneTex, Irvine, CA) and an EnVision+/HRP anti-rabbit secondary antibody (Agilent, Santa Clara, CA), with 3,3′-diaminobenzidine as the chromogen (340–07971; Dojin, Kumamoto, Japan). To distinguish staining patterns from hemosiderin pigment, Berlin blue staining was performed, followed by haematoxylin counterstaining. Images were captured using a SlideView VS200 (Evident, Tokyo, Japan) and processed with Olyvia software (Evident).

### Cytokine assay

Blood samples were collected from infected mice into EDTA-containing tubes at designated time points. Plasma was separated by centrifugation and stored at −80 °C until analysis. To inactivate DENV, plasma samples were mixed at a 1 : 2 ratio with Cell Lysing Buffer (Thermo Fisher Scientific, Waltham, MA, USA) prior to use. Cytokine concentrations in the inactivated plasma were measured using the Mouse Th1/Th2 10-plex FlowCytomix kit (eBioscience, San Diego, CA, USA) according to the manufacturer’s protocol. Flow cytometry was performed using a BD FACSCanto II (BD Biosciences, San Jose, CA, USA). The acquired data were analysed using FlowCytomix Pro software (version 2.3; eBioscience) to calculate cytokine concentrations based on standard curves generated from known calibrators. The analytes included IL-1α, IL-2, IL-4, IL-5, IL-6, IL-10, IL-17, IFN-γ, TNF-α and GM-CSF.

### Statistical analysis

Statistical analyses were performed using GraphPad Prism version 9.5.1 (GraphPad Software, San Diego, CA, USA). Survival curves were analysed using the log-rank (Mantel–Cox) test. Comparisons between groups were conducted using one-way or two-way ANOVA followed by Tukey’s multiple comparison test or unpaired Student’s t-test, as appropriate. A *P*-value<0.05 was considered statistically significant.

## Results

### Pathogenicity and ADE of DENV strains in *Ifnar1–Ifngr1* DKO mice

**Fig. 2.**
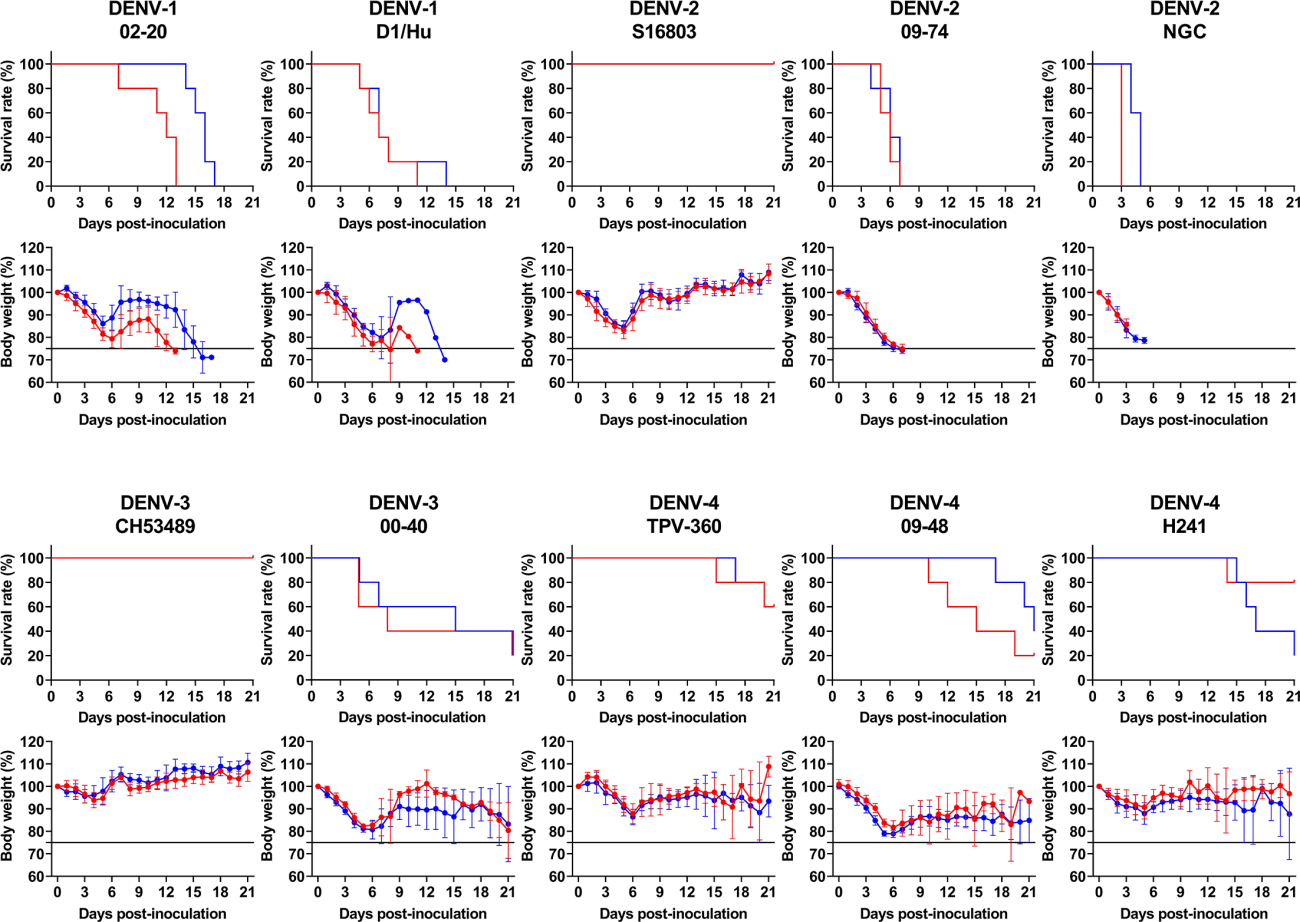
Survival and body weight changes in DENV-infected *Ifnar1–Ifngr1* DKO mice with or without 4G2 antibody treatment. *Ifnar1–Ifngr1* DKO mice (*n*=5 per group) were intraperitoneally inoculated with 1×10⁷ p.f.u. of the indicated DENV strains and pre-treated with the anti-DENV monoclonal antibody 4G2 (red symbols and lines) or PBS (blue symbols and lines) for ADE evaluation and comparative pathogenicity analyses. The upper panel shows survival curves, and the lower panel shows mean body weight changes±sd. Mice were euthanized upon reaching the endpoint, defined as a 25% loss of initial body weight (black line).

Although most human dengue infections are asymptomatic or mild, previous studies have established that *Ifnar1–Ifngr1* DKO mice, such as the AG129 model, preferentially develop severe disease [8,13, 23, 29]. To evaluate the pathogenicity of various DENV strains, we employed our newly developed C57BL/6J *Ifnar1–Ifngr1* DKO mouse model to analyse severe dengue-associated pathology [19]. To assess the potential for ADE, a subset of mice received the anti-DENV monoclonal antibody 4G2 1 day before viral inoculation. Body weight and survival were monitored daily to track disease progression and the effects of antibody treatment (Fig. 2).

For the DENV-1 02-20 strain, mice treated with the 4G2 antibody exhibited rapid weight loss, followed by a brief recovery and subsequent decline, with all animals requiring euthanasia by 13 dpi. In contrast, mice without antibody treatment experienced a slower rate of weight loss but still required euthanasia by 17 dpi. These results suggest that the presence of the antibody exacerbated disease severity, consistent with a possible ADE effect. The median lethal dose (LD_50_) for untreated mice was estimated to be ≤1×10¹ p.f.u., underscoring the pathogenic potential of this strain even in the absence of antibodies (Table 1).

**Table 1.** Dose-dependent survival outcomes of *Ifnar1–Ifngr1* DKO mice infected with DENV strains

Inoculation dose (p.f.u.)	10^6^	10^5^	10^4^	10^3^	10^2^	10^1^
**DENV-1 02-20** Deaths/total at 21 dpi Mortality (%)	5/5 100	5/5 100	5/5 100	4/5 80	3/5 60	3/5 60
**DENV-1 D1/Hu** Deaths/total at 21 dpi Mortality (%)	5/5 100	5/5 100	5/5 100	5/5 100	4/5 80	1/5 20
**DENV-2 09-74** Deaths/total at 21 dpi Mortality (%)	5/5 100	5/5 100	5/5 100	5/5 100	5/5 100	5/5 100
**DENV-2 NGC** Deaths/total at 21 dpi Mortality (%)	5/5 100	5/5 100	5/5 100	5/5 100	5/5 100	5/5 100

LD₅₀ values were estimated based on cumulative mortality assessed up to 21 dpi using the Reed–Muench method. For DENV-1 02-20, DENV-2 09-74 and DENV-2 NGC, cumulative mortality exceeded 50% at all tested doses down to 1×10¹ p.f.u.; therefore, the LD₅₀ values were estimated to be ≤1×10¹ p.f.u. under the experimental conditions tested. In contrast, for DENV-1 D1/Hu, dose-dependent mortality spanning the 50% threshold was observed, allowing calculation of an LD₅₀ value of ~3×10¹ p.f.u.

With the DENV-1 D1/Hu strain, both antibody-treated and untreated mice exhibited significant weight loss, with all animals requiring euthanasia by 14 dpi. No significant difference in disease progression was observed between the two groups, suggesting that the pathogenicity of this strain was high regardless of antibody presence. In untreated mice, dose-dependent mortality spanning the 50% threshold was observed (Table 1), allowing estimation of an LD₅₀ value of ~3×10¹ p.f.u. based on cumulative mortality assessed up to 21 dpi. These findings suggest that the inherent pathogenicity of the virus plays a more critical role than antibody-mediated effects in determining the disease outcome of this strain.

Mice infected with either DENV-2 09-74 or DENV-2 NGC exhibited severe weight loss, with both strains showing an estimated LD50 of ≤1×10^1^ p.f.u. (Table 1), and all animals requiring euthanasia by 7 dpi for DENV-2 09-74 and by 5 dpi for DENV-2 NGC. No significant difference was observed between the antibody-treated and untreated groups, suggesting that the high pathogenicity of these strains drives rapid disease progression independent of antibody presence. The severity of the infection indicates that the virus itself is the primary determinant of the rapid deterioration observed in mice, rather than any potential ADE effect. Conversely, mice infected with DENV-2 S16803 showed minimal weight changes and remained healthy throughout the study period, regardless of antibody treatment.

Compared with DENV-1 and DENV-2, DENV-3 and DENV-4 strains showed markedly lower virulence based on the LD_50_ in *Ifnar1–Ifngr1* DKO mice. With DENV-3 00-40, both the antibody-treated and PBS control groups had a survival rate of 20% at 21 dpi, indicating moderate virulence of the strain. Infection with DENV-3 CH53489 resulted in 100% survival in both groups, with minimal body weight changes throughout the observation period. In contrast, infection with certain DENV-4 strains revealed an influence of antibody administration on disease severity. With DENV-4 09-48, survival was 20% in the antibody-treated group compared to 40% in the control group, and the antibody-treated mice experienced more pronounced weight loss during the initial infection period. Similarly, DENV-4 H241 infection led to a survival rate of 20% in the antibody-treated group versus 80% in the control group, indicating a significant decrease in survival associated with antibody treatment. These findings suggest that, although DENV-3 and DENV-4 were generally less virulent than DENV-1 and DENV-2, certain DENV-4 strains caused enhanced disease severity and reduced survival rates in the presence of antibodies, possibly attributable to ADE.

### Pathogenicity and ADE of DENV strains in *Ifnar1*- and *Ifngr1*-single-KO mice

To further investigate host susceptibility and examine the role of IFN signalling, the same DENV strains were evaluated in single-KO mice (*Ifnar1* KO and *Ifngr1* KO) (Fig. S1, available in the online Supplementary Material). *Ifngr1*-KO mice exhibited no significant weight loss or mortality, and no strain-specific differences in virulence were observed regardless of antibody treatment, indicating that type I IFN signalling alone is sufficient to suppress viral replication and prevent clinical illness. In contrast, *Ifnar1*-KO mice exhibited increased susceptibility to infection with DENV-1 02-20 and DENV-2 09-74, including evidence of ADE, as reflected by greater body weight loss compared to infections with other strains. However, overall disease severity in these mice remained substantially lower than that in the DKO model. Ifnar1-KO mice exhibited moderate weight loss following infection but fully recovered, suggesting that intact type II IFN (IFN-γ) signalling can partially compensate for the absence of type I signalling and aid in viral control. Additionally, the absence of IFN-γ signalling may mitigate inflammatory damage that could contribute to tissue injury.

Collectively, these findings demonstrate a broad spectrum of DENV strain-specific pathogenicity in the DKO model, ranging from avirulent (DENV-2 S16803, DENV-3 CH53489) to highly lethal (DENV-1 D1/Hu, DENV-2 NGC). These results underscore the essential roles of both viral genetic factors and host immune pathways, including ADE, in driving disease progression.

### Clinical changes in *Ifnar1–Ifngr1* DKO mice infected with the DENV-1 02-20 strain

**Fig. 3.**
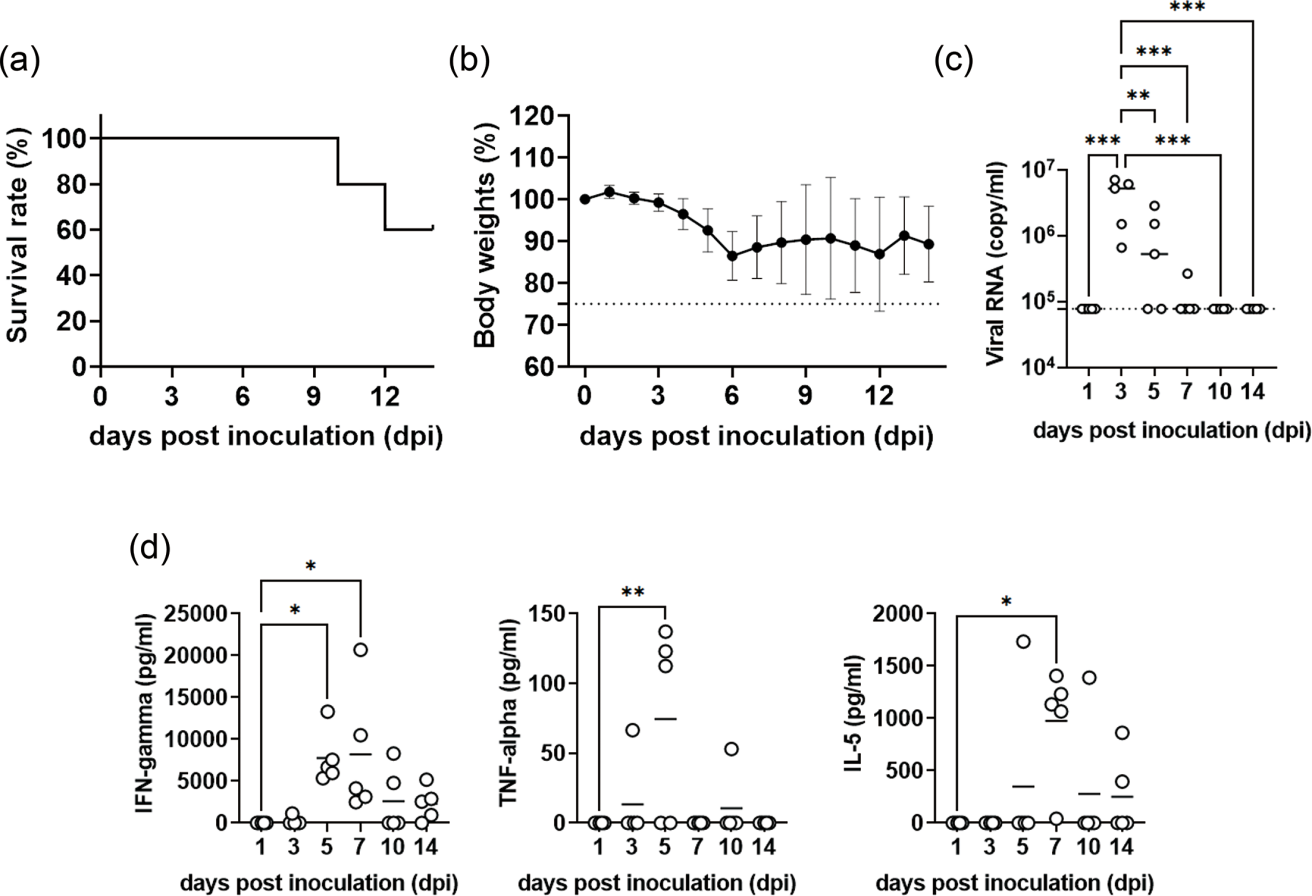
Clinical, virological, histological and cytokine dynamics in *Ifnar1–Ifngr1* DKO mice infected with DENV-1 02-20. (**a**) Survival rate and (**b**) body weight changes in mice intraperitoneally infected with 1×10⁵ p.f.u. (*n*=5). For virological, histological and cytokine analyses, independent cohorts of mice were euthanized at pre-specified time points (1, 3, 5, 7, 10 and 14 dpi; *n*=5 mice per time point). To ensure sufficient sample numbers at later time points despite disease-associated mortality, additional mice were included at the start of the experiment and allocated to scheduled euthanasia cohorts as needed. (**c**) Viral RNA in serum samples. (**d**) Serum cytokine levels (IFN-γ, TNF-α and IL-5) measured using a multiplex bead assay. Data are shown as mean±sd. Statistical significance was determined by one-way ANOVA (* p < 0.05, ** p < 0.01, *** p < 0.001, **** p < 0.0001).

To characterize the kinetics of DENV-1 infection in a highly susceptible host, *Ifnar1–Ifngr1* DKO mice were inoculated intraperitoneally with 1×10^5^ p.f.u. of DENV-1 02-20 and euthanized at 1, 3, 5, 7, 10 and 14 dpi (*n*=5 per time point) (Fig. 3a, b). At 1 and 3 dpi, no clinical or histopathological abnormalities were observed (Fig. S2). At 3 dpi, the mean plasma viral load reached 4.1×10^6^ copies per millilitre (Fig. 3c), indicating robust early viraemia.

**Fig. 4.**
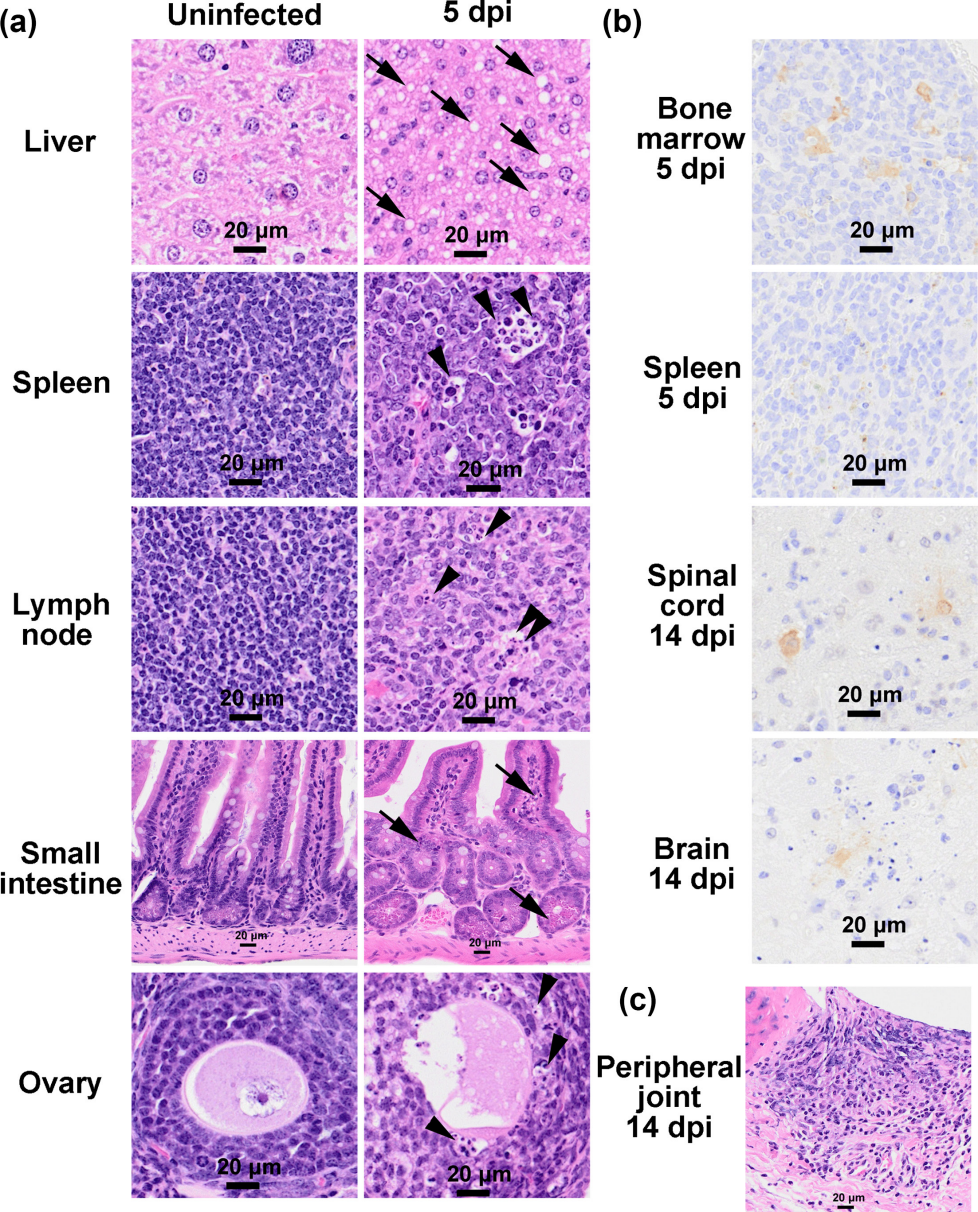
Histopathological and immunohistochemical features of DENV infection in *Ifnar1–Ifngr1* DKO mice infected with DENV-1 02-20. (**a**) Representative HE-stained sections of liver, spleen, lymph node, small intestine and ovary from uninfected control mice and DENV-infected mice at 5 dpi. Arrows indicate pronounced vacuolar degeneration in the liver and apoptotic cells sloughed into the crypt spaces in the small intestine. Arrowheads indicate cells with fragmented nuclei, consistent with apoptotic morphology in the spleen, lymph node and ovary. (**b**) Immunohistochemical detection of DENV NS3 antigen in bone marrow and spleen at 5 dpi, and in spinal cord and brain at 14 dpi. (**c**) Representative HE-stained sections of peripheral joints from DENV-infected mice at 14 dpi, showing inflammatory changes in periarticular tissues and synovial regions. All images are representative of infected mice at the indicated time points.

A single pathological peak was observed at 5 dpi. Mice exhibited their first body weight nadir (mean loss≈10%). Histologically, pronounced swelling and vacuolar degeneration in hepatocytes, increased apoptosis of lymphocytes in the spleen and lymph nodes, epithelial apoptosis and sloughing into the crypt spaces in the small intestine and apoptosis of granulosa cells in the ovary were observed, suggesting systemic cellular damage (Fig. 4a).

Viral antigen (NS3) distribution, assessed via immunohistochemistry, revealed widespread dissemination at 5 dpi (Table 2). In the femoral bone marrow, viral antigen was detected in resident macrophages from 3 dpi, peaked at 5 dpi, and was absent in most mice by 7 dpi (Fig. 3d). Weak-to-moderate (+ to ++) expression was observed in macrophages in the spleen, mesenteric lymph nodes, uterus, ovary and omentum and throughout the gastrointestinal tract, including the stomach and intestines.

**Table 2.** Distribution of NS3 viral antigen–positive tissues in DENV-1 02-20-infected *Ifnar1–Ifngr1* DKO mice. No pathological changes were detected in the heart, lung, liver, kidney, pancreas, small intestine, large intestine, mesenteric lymph node or bladder. The (+) represents the number of positive cells per 10 HPFs (1 HPF=0.26 mm²): (+), 1 to 19 positive cells; (++), 20 to 99 positive cells; (+++), ≥100 positive cells

Days post-infection (dpi)	3 dpi					5 dpi					7 dpi					10 dpi					14 dpi				
Animal no.	**1**	**2**	**3**	**4**	**5**	**1**	**2**	**3**	**4**	**5**	**1**	**2**	**3**	**4**	**5**	**1**	**2**	**3**	**4**	**5**	**1**	**2**	**3**	**4**	**5**
**Brain**																	**++**				**++**	**++**	**+**	**+**	**++**
**Spleen**	**+**					**+**	**+**	**+**																	
**Stomach**								**+**	**+**																
**Uterus**						**+**	**+**	**+**	**+**	**+**															
**Ovary**						**+**	**+**	**+**	**+**	**+**			**+**												
**Spinal cord**																**+**	**++**				**++**	**+**	**++**	**++**	**++**
**Femoral bone marrow**	**+**	**+**	**+**	**+**	**+**	**+++**	**+++**	**+++**	**+++**	**++**			**++**					**+**							

Notably, spinal cord involvement was first detected at 10 dpi and persisted through 14 dpi, whereas brain involvement became evident at 10 dpi and intensified by 14 dpi. These findings indicate a systemic infection that subsequently progresses to central nervous system involvement (Fig. 4b).

Multiplex cytokine profiling revealed synchronous peaks of IFN-γ (~8×10^3^ pg ml^−1^), IL-5 (~1×10^3^ pg ml^−1^) and TNF-α (~7.5×10^1^ pg ml^−1^; observed in only three out of five mice) between 5 and 7 dpi (Fig. 3d). By 10 dpi, both lesions and cytokine concentrations had declined sharply, viral antigen was scarce, and body weight had begun to recover. However, given that IFN-γ is unlikely to contribute to tissue damage in DKO mice due to the absence of its receptor and that TNF-α elevation occurred only in a subset of animals, a direct link between these cytokines and the pathological findings remains unclear. Therefore, although the cytokine response temporally overlapped with systemic injury, its role in driving pathology remains uncertain.

Although no mortality was observed before 10 dpi, a delayed phase of morbidity became evident between 10 and 14 dpi. At 10 dpi, early encephalomyelitis – characterized by viral antigen–positive neurons – was evident. By 14 dpi, inflammatory changes were occasionally observed in peripheral joints (Fig. 4c). By 14 dpi, three out of five animals exhibited severe lethargy, hindlimb weakness and a second precipitous weight-loss episode (>20% of baseline; Fig. 3b), necessitating humane euthanasia.

These findings indicate a biphasic disease course in DENV-1 02-20–infected *Ifnar1–Ifngr1* DKO mice. An early systemic phase peaked at 5 dpi, driven by high-level viraemia (up to 4.1×10^6^ copies per millilitre at 3 dpi) and a transient IFN-γ/IL-5/TNF-α response, which precipitated hepatocellular vacuolation, gastrointestinal epithelial loss and the first weight-loss trough. A subsequent neuroarthritogenic phase (≥10 dpi) involved a combination of direct viral cytopathology in the central nervous system and immune-mediated inflammatory changes in peripheral joints, leading to late clinical deterioration and death in a subset of mice.

## Discussion

The study demonstrates substantial variability in the pathogenicity of various DENV strains in our newly developed *Ifnar1–Ifngr1* DKO mouse model. The heightened susceptibility of these mice enables robust assessment of viral virulence and ADE under highly permissive conditions, supporting the utility of this model for mechanistic studies of DENV infection. Notably, the DENV-1 02-20 strain exhibited a significant ADE, as mice treated with the anti-DENV 4G2 antibody developed more severe disease than untreated mice. These findings indicate that antibodies can exacerbate infection with certain DENV strains, resulting in increased disease severity within this mouse model. In contrast, strains such as DENV-1 D1/Hu, DENV-2 09-74 and DENV-2 NGC caused severe disease irrespective of antibody administration, suggesting that, under the experimental conditions used here, including high inoculation doses and rapid disease progression, potential antibody-mediated effects were not readily observable. It should be noted that this model does not recapitulate key features of human dengue infection, which is predominantly asymptomatic and rarely involves the central nervous system. Therefore, the present model should be regarded as a tool for dissecting virus- and antibody-dependent pathogenic mechanisms under highly permissive conditions, rather than as a direct representation of the full spectrum of human dengue disease.

The differential pathogenicity observed among the DENV strains underscores the importance of strain-specific viral characteristics in driving disease progression. The severe outcomes associated with DENV-1 D1/Hu and the highly virulent DENV-2 strains suggest that these viruses possess genetic or phenotypic traits that enable rapid replication and dissemination, thereby overwhelming the host immune response, even before the eventual involvement of ADE. Conversely, the potential ADE observed with the DENV-1 02-20 strain is consistent with previous findings that infection by certain DENV serotypes and genotypes is more prone to ADE [18,30]. Collectively, these findings highlight the complex interplay between viral genetics and host immune responses in determining disease severity.

In addition to viral factors, our comparative analysis of KO mouse strains provides insights into the host immune mechanisms that modulate disease outcomes. Although *Ifnar1*-KO mice lack type I IFN signalling, they developed only mild disease following DENV-1 infection. This finding suggests that IFN-γ signalling may play a compensatory role in antiviral defence in these animals. In contrast, *Ifngr1*-KO mice – which retain intact type I IFN signalling but lack IFN-γ responsiveness – remained asymptomatic, indicating that type I IFNs alone are sufficient for viral control and that the absence of IFN-γ signalling may protect against immune-mediated tissue damage.

Among the DENV strains examined in this study, DENV-1 02-20 was selected for in-depth analyses because it uniquely allowed simultaneous evaluation of strain-specific virulence, IFN-dependent host susceptibility and ADE. This strain caused lethal disease in *Ifnar1–Ifngr1* DKO. In contrast, in *Ifnar1*-KO mice, the virus was not lethal on its own but caused 100% mortality only when mice were previously treated with the 4G2 antibody (ADE conditions). Importantly, DENV-1 02-20 consistently exhibited a pronounced ADE phenotype, which remains a central unresolved issue in dengue pathogenesis and vaccine development [18].

In contrast, strains such as DENV-2 S16803 and DENV-3 CH53489 showed minimal clinical manifestations and high survival rates even in the DKO model, suggesting intrinsically lower virulence under these experimental conditions. To date, *in vivo* infection studies of these strains have not been reported in AG129 mice, and therefore, direct comparisons across models are not currently possible. Nevertheless, previous AG129-based studies using other strains, most notably DENV-2 NGC, have demonstrated that DENV pathogenicity varies substantially depending on viral strain and experimental context [8,14, 31].

In contrast to AG129 mice, which are maintained on a 129/Sv genetic background and have primarily been used with a limited number of mouse-adapted DENV strains such as DENV-2 NGC [31,32], the *Ifnar1–Ifngr1* DKO mice used in this study are on a C57BL/6J background and permit direct comparison of multiple non–mouse-adapted clinical DENV isolates under identical experimental conditions. The genetic background, along with the differences in viral strains, represents an important distinction between the two models. Moreover, the C57BL/6J background is widely used in immunological research and offers greater compatibility with genetically engineered mouse lines. This facilitates future mechanistic studies involving additional immune modifications and improves reproducibility across laboratories. Notably, following intraperitoneal infection with DENV-2 NGC, *Ifnar1–Ifngr1* DKO mice reached humane endpoints at doses as low as 10²–10³ p.f.u., with marked body weight loss occurring as early as 5–7 dpi, whereas previous AG129-based studies reported prolonged survival at comparable doses [31,32]. This observation suggests a modestly higher susceptibility of the DKO model under these experimental conditions, rather than a qualitative difference in disease mechanisms. This distinction enables more systematic evaluation of strain-specific virulence and ADE phenotypes without reliance on virus adaptation.

Furthermore, in the developed *Ifnar1–Ifngr1* DKO model, we observed viraemia, elevated cytokine levels – including IFN-γ, IL-5 and TNF-α – and antigen presence in the bone marrow in DENV-infected *Ifnar1–Ifngr1* DKO mice without antibody treatment. These results are consistent with findings from other mouse models, such as AG129, A129 and C57BL/6J-based models, thereby validating the DKO model’s susceptibility and confirming its suitability for studying DENV infection [13,23, 29]. A previous study demonstrated that treatment with a neutralizing anti-TNF-α antibody prolonged survival and reduced liver damage in a DENV mouse model, suggesting a potential pathogenic role for TNF-α [33]. In our study, however, TNF-α elevation at 5 dpi was observed in only three out of five mice, limiting interpretability and restricting definitive conclusions regarding its contribution to disease progression. Although IFN-γ, TNF-α and IL-5 showed transient increases, the absence of IFN-γ signalling, along with the inconsistent elevation of TNF-α and IL-5, suggests that these cytokines are unlikely to be the primary mediators of systemic pathology. Therefore, while cytokine elevations coincided temporally with disease onset, a direct causal relationship between specific cytokines and tissue injury could not be established. Further studies are warranted to clarify the role of these immune mediators in DENV pathogenesis.

An isotype-matched control antibody was not included in the present study, which represents a limitation. PBS was used as a control to distinguish antibody-dependent effects from antibody-independent viral pathogenicity. Although the inclusion of an isotype control would have been ideal, previous ADE studies have demonstrated that non-specific IgG does not reproduce the enhancement observed with 4G2 [20,34]. Therefore, while future studies incorporating isotype controls will be important for mechanistic refinement, the qualitative conclusion that 4G2 enhances disease severity in this model is unlikely to be altered.

Our results have important implications for dengue vaccine development and therapeutic strategies. The potential for ADE, particularly with specific strains such as DENV-1 02-20, underscores the need for vaccines that elicit balanced, protective immune responses without increasing the risk of infection [4]. Vaccine candidates must be rigorously evaluated for their potential to induce ADE, particularly in populations with pre-existing immunity to various DENV serotypes. In this context, the *Ifnar1–Ifngr1* DKO model, despite its immunological limitations, provides a practical platform for assessing ADE-related risks during dengue vaccine development.

Understanding the mechanisms underlying the inherent virulence of certain DENV strains is essential for developing targeted antiviral therapies. The high pathogenicity of strains such as DENV-2 09-74 and DENV-2 NGC, with no apparent influence of antibody treatment under the conditions tested, suggests that interventions aimed at inhibiting viral replication or modulating host factors critical to the viral life cycle could be effective [7]. Additionally, dissecting the dual role of IFN-γ – as both a mediator of antiviral protection and a driver of immune pathology – may reveal novel therapeutic targets to mitigate disease severity while preserving host defence.

Overall, this study presents a cost-effective *Ifnar1–Ifngr1* DKO mouse model, on the C57BL/6J background, that exhibits high susceptibility to DENV infection and captures several key pathological features observed in severe dengue cases, rather than the full clinical spectrum of human dengue infection, including ADE, strain-specific virulence, and immune-mediated pathology. We acknowledge that the *Ifnar1–Ifngr1* DKO model does not reproduce the high frequency of asymptomatic or mild dengue observed in humans. Instead, this model preferentially manifests severe disease requiring humane endpoints and should therefore be regarded as a model of severe dengue rather than of average human infection. This model will support future research aimed at identifying viral genetic determinants of virulence and ADE, as well as further characterizing host immune responses, including cytokine dynamics and cellular activation. Such insights are key for developing more effective vaccines and therapies that address the complex interplay between DENV and the immune system. Clarifying the specific contributions of the IFN-α/β and IFN-γ pathways to viral clearance versus tissue damage will be particularly important.

## Supplementary material

10.1099/jgv.0.002267 Uncited Supplementary Material 1.

## Abbreviations

ADE: antibody-dependent enhancement
DENV: dengue virus
DKO: double-knockout
dpi: days post-infection
HE: haematoxylin and eosin
KO: knockout
NGC: New Guinea C
qRT-PCR: quantitative reverse transcription PCR
