# Supplementary material for: Strain-specific pathogenicity and antibody-dependent enhancement of dengue virus infection in Ifnar1–Ifngr1 double-knockout mice
Source: J Gen Virol. 2026 May 18;107(5):002267. doi: 10.1099/jgv.0.002267 (PMC13192526; doi:10.1099/jgv.0.002267)
Supplement: Uncited Supplementary Material 1. [file jgv-107-02267-s001.pdf]

Supporting Information

Strain-specific pathogenicity and antibody-dependent enhancement of dengue virus infection in *Ifnar1–Ifngr1* double-knockout mice

Author names

Takayuki Hishiki[1,†], Milagros Virhuez-Mendoza[2,†], Yusuke Sakai[3], Akihiko Uda[2,*], Seiya Ozono[3], Yuko Sato[3], Sorane Machiya[2], Kotomi Sato[2], Yuta Utsui[2], Shigeru Tajima[4], Chang-Kweng Lim[4], Akitoyo Hotta[2,7], Yudai Kuroda[2], Tsukasa Yamamoto[2], Yoshimasa Takahashi[5], Tadaki Suzuki[3,6], Koichi Watashi[1] and Ken Maeda[2]

Affiliation(s)

[1]Department of Drug Development, National Institute of Infectious Diseases, Japan Institute for Health Security, Shinjuku, Tokyo, Japan.

[2]Department of Veterinary Science, National Institute of Infectious Diseases, Japan Institute for Health Security, Shinjuku, Tokyo, Japan.

[3]Department of Infectious Disease Pathology, National Institute of Infectious Diseases, Japan Institute for Health Security, Shinjuku, Tokyo, Japan.

[4]Department of Virology I, National Institute of Infectious Diseases, Japan Institute for Health Security, Shinjuku, Tokyo, Japan.

[5]Research Center for Vaccine Development, National Institute of Infectious Diseases, Japan Institute for Health Security, Shinjuku, Tokyo, Japan.

[6]Department of Infectious Disease Pathobiology, Graduate School of Medicine, Chiba University, Chiba, Japan.

[7]Laboratory of Bacteria Collection Bank, Research Center for Biosafety, Laboratory Animal and Pathogen Bank, National Institute of Infectious Diseases, Japan Institute for Health Security, Shinjuku, Tokyo, Japan.

†These authors contributed equally to this work.

Supporting Materials and Methods

Figure S1, S2,

SUPPORTING MATERIALS and METHODS

For LD_{50} determination, mice were infected with lower viral doses as described in the main Materials and Methods section.

Animal experiments and viral quantification

For antibody-dependent enhancement (ADE) evaluation and comparative pathogenicity analyses, higher viral doses (1×10^7 PFU) were used and were not included in LD_{50} calculations. To evaluate the influence of antibodies on disease progression, a subset of mice was administered the anti-DENV monoclonal antibody 4G2 (clone 4G2; mouse IgG2a), originally generated in mice and obtained from ATCC (HB-112), at a dose of 50 µg per mouse in 100 µL PBS one day prior to viral inoculation. The dose was selected based on previous ADE studies demonstrating robust enhancement at comparable antibody concentrations [1, 2]. Control mice received PBS alone. Mice were euthanized when body weight loss exceeded 25% of the initial body weight, in accordance with the approved euthanasia criteria (25% loss of initial body weight).

Supplementary Figure S1

A

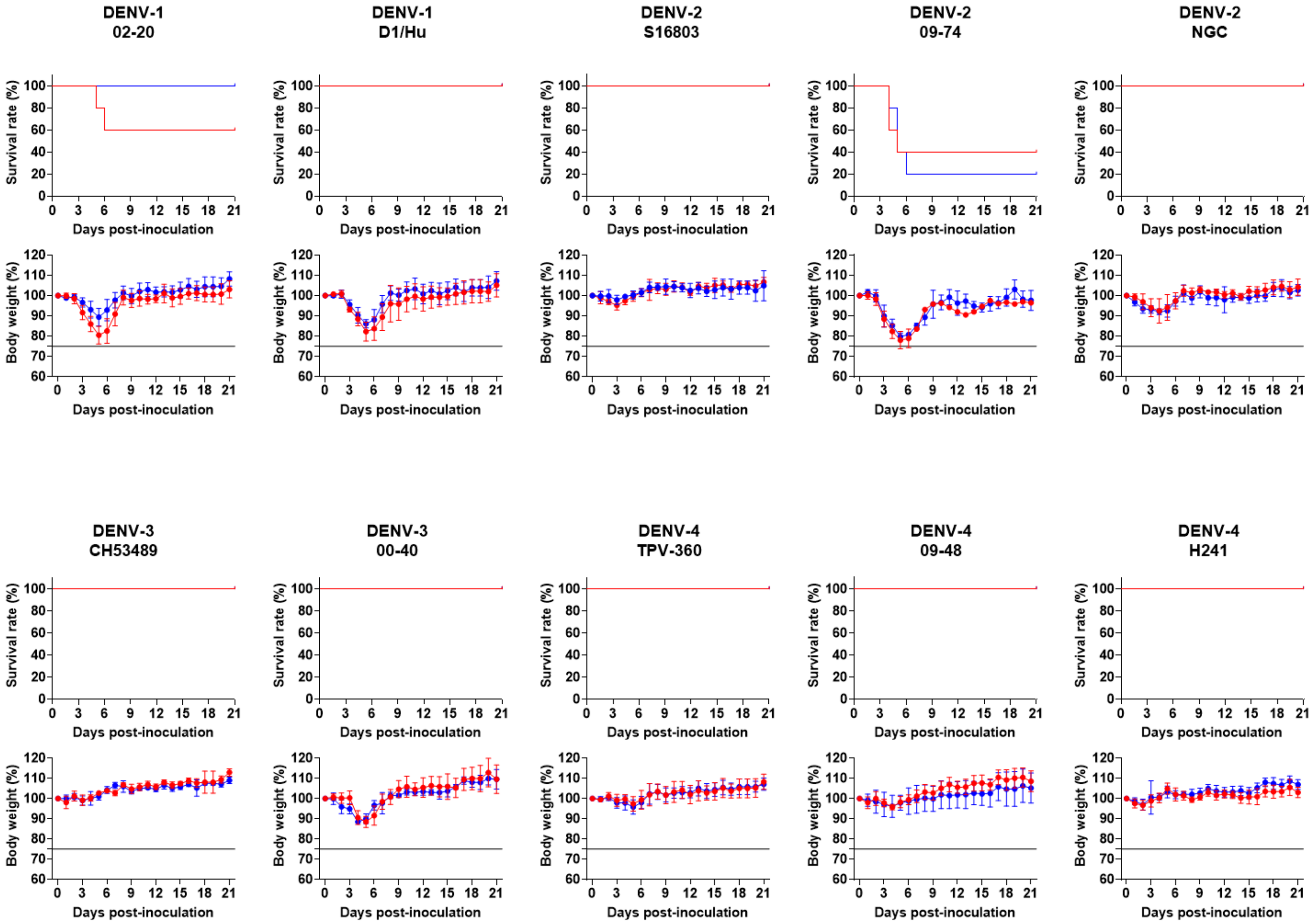

Supplementary Figure S1

B

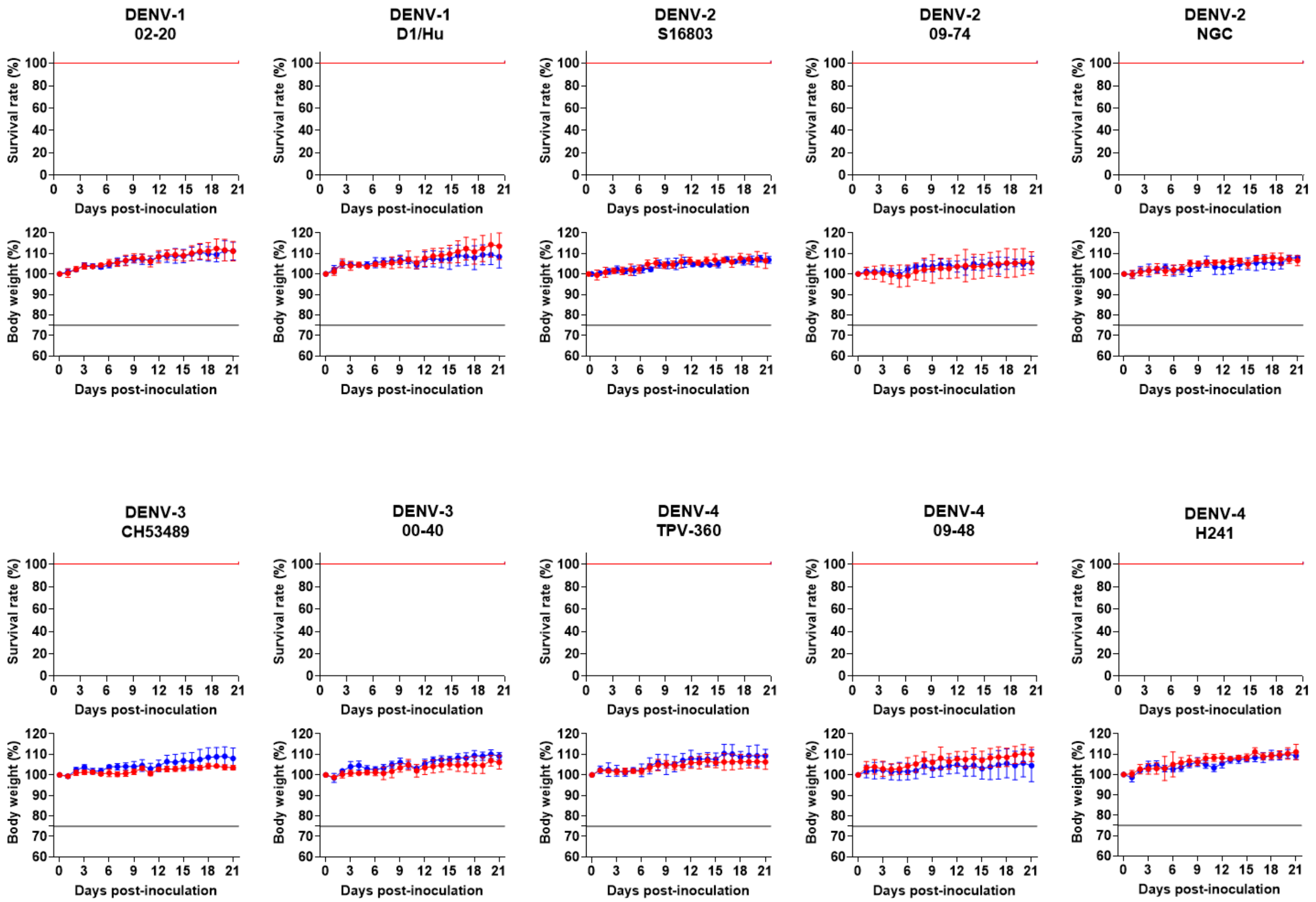

Supplementary Fig. S1. Survival and body weight changes in dengue virus (DENV)-infected Ifnar1 KO mice and Ifngr1 KO mice with or without 4G2 antibody treatment. Ifnar1 KO mice (Figure A) and Ifngr1 KO mice (Figure B) were intraperitoneally inoculated (n = 5 per group) with 1×10^7 plaque-forming units (PFU) of the indicated DENV strains and pre-treated with the anti-DENV monoclonal antibody 4G2 (red symbols and lines) or PBS (blue symbols and lines) for antibody-dependent enhancement (ADE) evaluation and comparative pathogenicity analyses. The upper panel shows survival curves, and the lower panel shows mean body weight changes ± standard deviation (SD). Mice were euthanized upon reaching the endpoint, defined as a 25% loss of initial body weight (black line).

Supplementary Figure S2

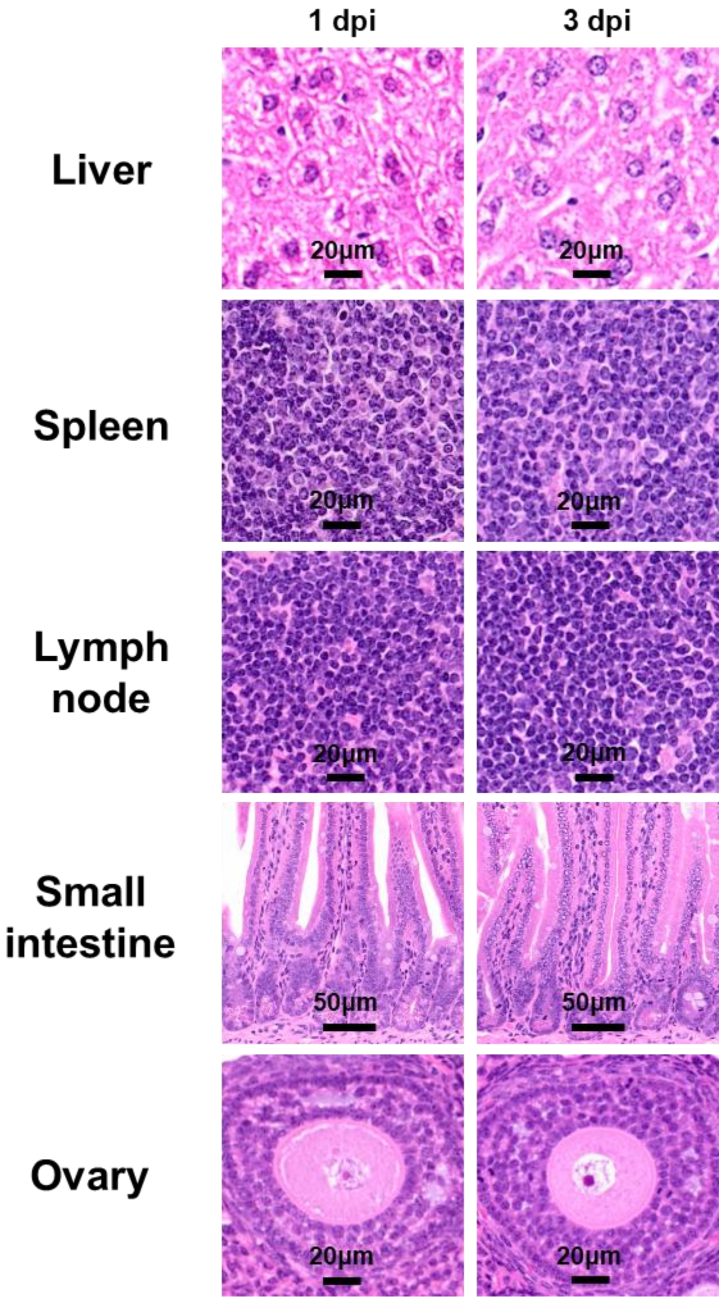

Supplementary Fig. S2. Histopathological features of early DENV infection in *Ifnar1-Ifngr1* DKO mice infected with DENV-1 strain 02-20. Representative hematoxylin and eosin (HE)-stained sections of liver, spleen, lymph node, small intestine, and ovary from DKO mice at 1 and 3 days post-infection (dpi). No histopathological abnormalities were observed in any of the tissues examined at 1 dpi (left panels) and 3 dpi (right panels). Scale bars: 20 μm (liver, spleen, lymph node, ovary) and 50 μm (small intestine).

References

1. **Watanabe S, Chan KW, Dow G, Ooi EE, Low JG et al.** Optimizing celgosivir therapy in mouse models of dengue virus infection of serotypes 1 and 2: The search for a window for potential therapeutic efficacy. *Antiviral Res* 2016;127:10-19.
2. **Watanabe S, Tan KH, Rathore AP, Rozen-Gagnon K, Shuai W et al.** The magnitude of dengue virus NS1 protein secretion is strain dependent and does not correlate with severe pathologies in the mouse infection model. *J Virol* 2012;86(10):5508-5514.
